# A case of right fronto-parietal gliosarcoma

**DOI:** 10.1016/j.radcr.2024.11.036

**Published:** 2024-12-03

**Authors:** Na Li, Wangsheng Chen

**Affiliations:** Affiliated Hainan Hospital of Hainan Medical University (Hainan General Hospital ), NO. 19, XiuHua ST, XiuYing District, Haikou, Hainan, 570311, PR China

**Keywords:** Intracranial tumor, Gliosarcoma, Right fronto-parietal lobe, Magnetic resonance imaging

## Abstract

Gliosarcoma (GS) is a rare subtype of glioblastoma multiforme, characterized by a shorter clinical course and poorer prognosis compared to glioblastoma. Here, we report the case of a 50-year-old male patient who presented with episodic loss of consciousness and left-sided limb weakness for one month. MRI revealed a complex neoplastic lesion in the right fronto-parietal region. Postoperative pathology confirmed GS, and the patient underwent adjuvant radiotherapy and chemotherapy. This case highlights the characteristic features of GS through a combination of imaging and pathological findings, providing valuable insights for radiologists.

## Introduction

Gliosarcoma (GS) is a rare and highly aggressive malignant tumor, with a poorer prognosis compared to glioblastoma multiforme (GBM) [1]. The radiological features of GS often overlap with those of GBM, complicating diagnosis. In this report, we present a case of GS diagnosed through pathological examination. We describe the clinical and radiological characteristics of this condition and discuss the associated diagnostic challenges, differential diagnoses, treatment strategies, and therapeutic challenges. Additionally, we provide a comprehensive literature review to enhance understanding and facilitate early diagnosis of GS.

## Case presentation

**Fig. 1:**
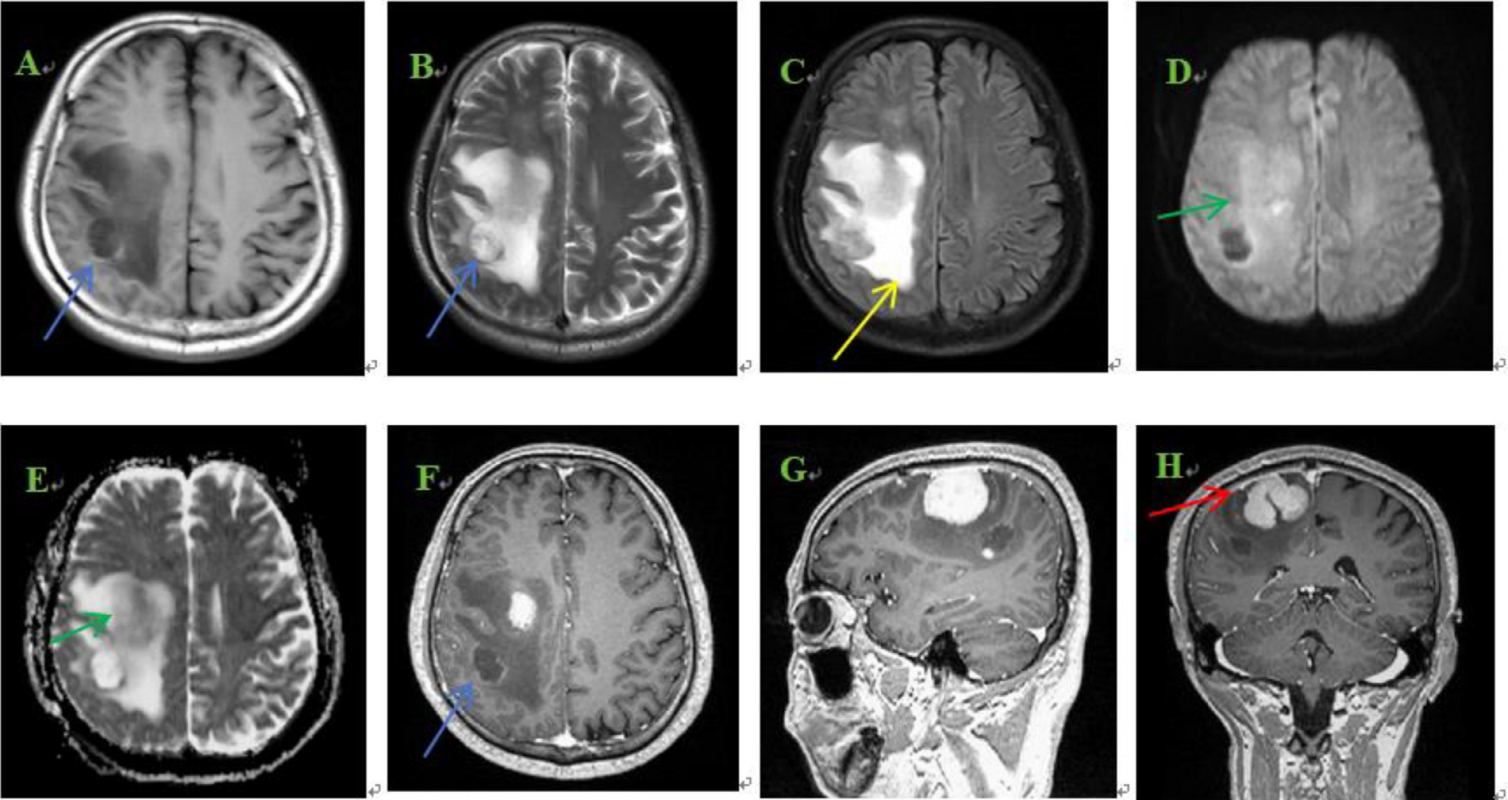
MRI images. (A-E) Axial T1WI, T2WI, T2-FLAIR, DWI, and ADC images, respectively; (F-H) Axial and sagittal contrast-enhanced scans. The images reveal nodular and mass-like lesions in the right fronto-parietal lobe, displaying hypointense T1 and slightly hyperintense T2 signals, with extensive peritumoral edema (yellow arrow). Contrast-enhanced images show significant enhancement, with cystic and necrotic areas visible within the lesion (blue arrow). There is a clear mass effect and infiltration of the adjacent dura mater (red arrow). On DWI, part of the lesion exhibits mild diffusion restriction (green arrow).

**Fig. 2:**
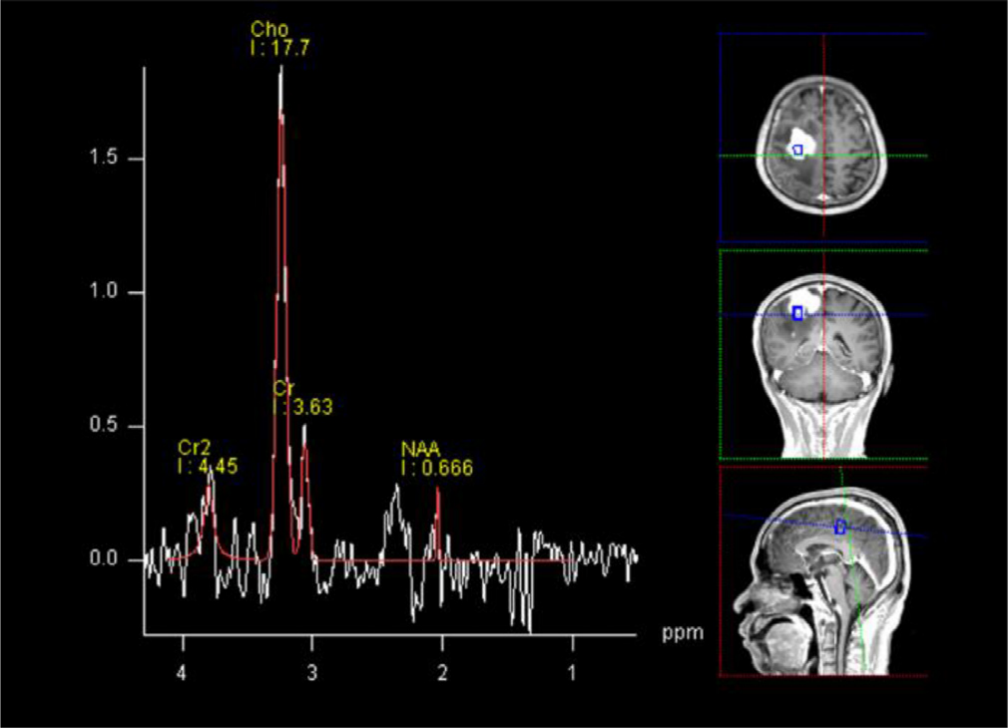
Magnetic resonance spectroscopy (MRS). MRS shows a marked elevation of the Cho peak and a reduction in the NAA peak in the right frontal lesion. The Cho/NAA ratio is 26.57, the NAA/Cr ratio is 0.18, and the Cho/Cr ratio is 4.87, indicating neuronal cell damage.

**Fig. 3:**
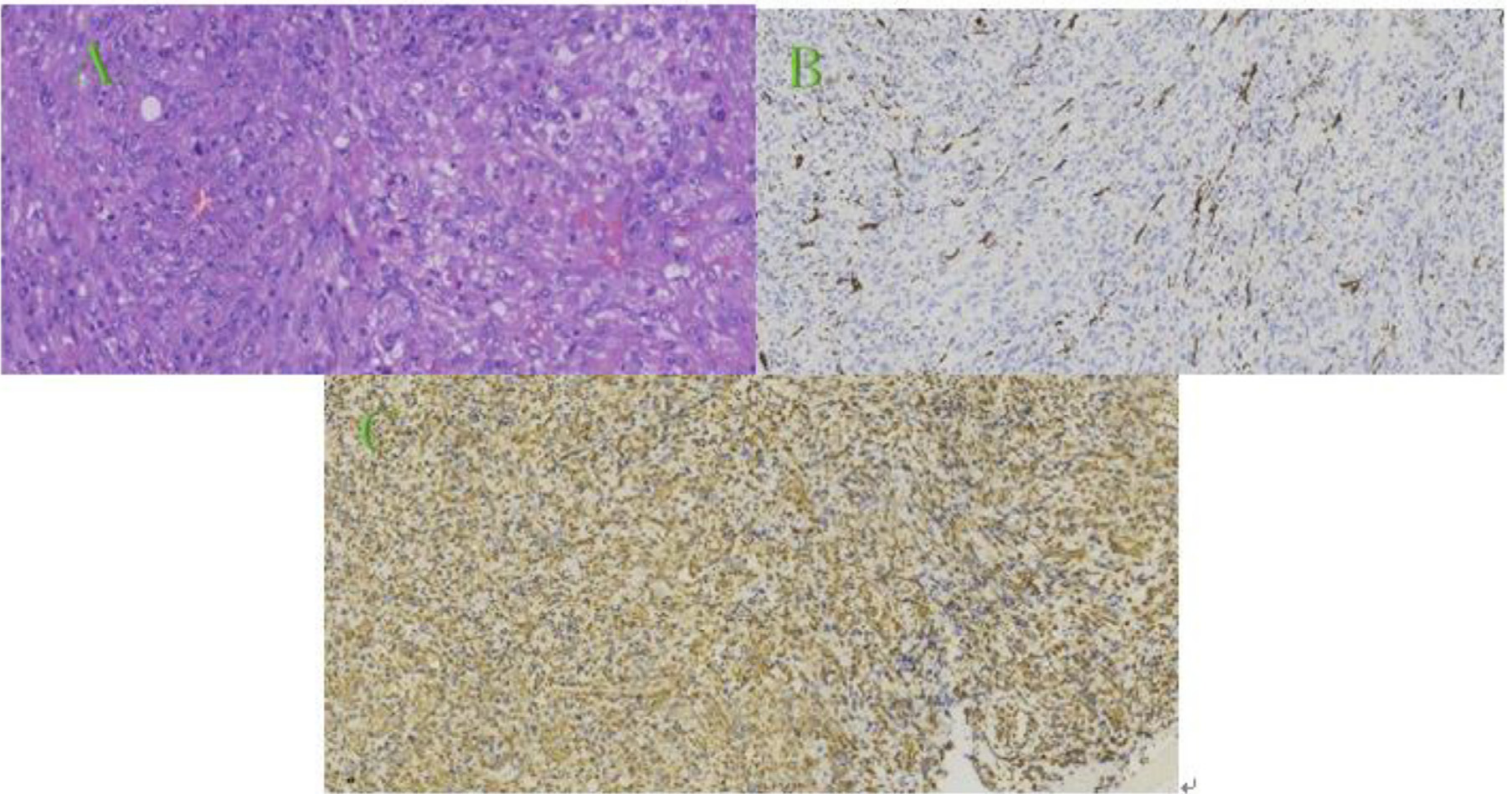
Pathological examination. (A) Tumor cells are diffusely arranged in sheets, exhibiting spindle or ovoid shapes with significant nuclear atypia, some with eosinophilic nucleoli, and frequent mitotic figures (HE, × 40); (B) Immunohistochemical staining: Tumor cells are focally positive for GFAP (× 20); (C) Immunohistochemical staining: Tumor cells are positive for Vim (× 20).

The patient is a 50-year-old male who initially presented with intermittent convulsions of the left upper limb, primarily affecting the left forearm, 20 days prior to admission. These episodes were not given much attention. Fourteen days before hospitalization, he developed weakness in the left upper limb, characterized by an unstable grip, while his gait remained relatively unaffected, though his left lower limb exhibited a slight drag. The patient sought care at an outside hospital, where a neurological examination revealed a left-sided muscle strength of 5 and a right-sided muscle strength of 5. Hyperreflexia of the left knee tendon was observed. Neuroimaging identified a tumor in the right fronto-parietal region. Laboratory results showed elevated levels of cerebrospinal fluid/24-hour urine total protein (503.90 mg/L; normal range 150-450 mg/L), glucose (6.51 mmol/L; normal range 2.5-4.8 mmol/L), and immunoglobulin M (0.172 g/L; normal range 0-0.013 g/L). Multiple tumor markers, including carbohydrate antigen 199, neuron-specific enolase, prostate-specific antigen (PSA), squamous cell carcinoma antigen, free PSA, alpha-fetoprotein (AFP), and carcinoembryonic antigen (CEA), were within normal limits. No treatment was initiated at the time, but the patient and his family opted for further evaluation and treatment at our institution. Upon admission, the patient was conscious but presented with poor mental status, appetite, and sleep, along with weight loss. His medical history included hypertension, diabetes mellitus, and previous colonic polyp resection. He also had a family history of diabetes. Magnetic resonance imaging (MRI) revealed multiple nodular and mass-like abnormal signals in the right fronto-parietal region, with the largest lesion located in the right frontal lobe, measuring approximately 4.6 × 3.5 cm. The lesion exhibited hypointense signals on T1-weighted images and slightly hyperintense signals on T2-weighted images. Diffusion-weighted imaging (DWI) showed mild restricted diffusion in some parts of the lesion. Extensive surrounding edema was noted in the right frontal lobe. Contrast-enhanced MRI revealed significant heterogeneous enhancement of the lesion, with non-enhancing cystic and necrotic areas (Fig. 1). Magnetic resonance spectroscopy (MRS) demonstrated a markedly elevated Cho peak and decreased NAA peak in the right frontal lesion, with a Cho/NAA ratio of 26.57, NAA/Cr ratio of 0.18, and Cho/Cr ratio of 4.87 (Fig. 2). The patient underwent surgical resection of the right fronto-parietal mass, and pathological examination confirmed the diagnosis of GS (WHO grade IV) (Fig. 3).

During craniotomy, the tumor measured approximately 4.5 × 4.5 × 4.0 cm, appearing reddish in color, solid, with a medium to firm texture, and highly vascularized. The tumor displayed clear demarcation from the surrounding normal brain tissue but was tightly adhered to the central sulcus vein and precentral sulcus vein. Significant edema was observed in the surrounding brain tissue. Intraoperative biopsy revealed tumor cells arranged in sheets or fascicles, with focal microcystic structures. The tumor cells were spindle-shaped or ovoid, with some displaying vacuolated nuclei, prominent eosinophilic nucleoli, and frequent mitotic figures. The stroma showed a sparse infiltration of lymphocytes, and focal areas of necrosis were noted. Immunohistochemical staining results were as follows: IDH1 (-), Ki-67 (+, 10%), EGFR (2+), GFAP (focal +), Olig-2 (few cells +), P53 (weak +), ATRX (+), EMA (-), PR (-), MelanA (weak +), HMB45 (-), MGMT (+), CD34 (-), Vim (+), CK (-). The pathological diagnosis confirmed GS (WHO grade IV) (Fig. 3).

On postoperative day 2, the patient developed cerebral edema, increased intracranial pressure, and brain herniation, necessitating decompressive craniectomy. Postoperatively, the patient received adjuvant treatment with temozolomide and radiotherapy. Although occasional seizures occurred, his clinical course remained relatively stable. However, during routine follow-up, an abnormally enhancing nodule was detected at the junction of the right fronto-parietal region, extending to the genu of the corpus callosum, suggestive of recurrence. Despite this, the patient and his family declined further treatment.

## Discussion

In the 2016 WHO classification of central nervous system tumors, GS was classified as a subtype of IDH-wildtype GBM [2]. However, in the 2021 classification, GS was no longer recognized as an independent subtype [3]. This reflects the current uncertainty regarding its biological and molecular characteristics, with its diagnosis still largely relying on histopathological evaluation. Due to the overlap in radiological features with other intracranial tumors, differentiation from other entities remains challenging.

GS has a low incidence, accounting for 1.8% to 8% of GBM cases [4]. The age of onset ranges from 4 to 84 years, with a median age of 52 years, and the male-to-female ratio is approximately 1.4-1.8:1 [5]. In this case, the patient's age of 50 years is consistent with previous reports. The primary symptoms include seizures, headaches often accompanied by vomiting, and focal neurological deficits, which are nonspecific and typically related to tumor location and size. According to the literature, extracranial metastasis occurs in approximately 11% of GS cases, accounting for more than one-third of all metastatic gliomas. However, in this case, no extracranial metastasis was observed. Supratentorial lesions are most common, with the lungs, liver, and lymph nodes being frequent metastatic sites [6,7].

Most GSs are located in the supratentorial cerebral hemispheres, with the temporal lobe being the most commonly affected region [8]. Han et al. [9] reported that these tumors often presented with irregular shapes. On MRI, GSs typically appear as hypointense on T1-weighted images and exhibit mixed hyperintensity or hyperintensity on T2-weighted images. Contrast-enhanced imaging shows significant enhancement, and necrotic or cystic areas may exhibit heterogeneous or ring-like enhancement. Peritumoral edema and mass effect are prominent. Most GSs have well-defined margins, but in some cases, they are adherent to the dura, leading to potential misdiagnosis as meningioma [10].

GS is characterized histopathologically by 2 distinct components: a gliomatous region, positive for hematoxylin-eosin (HE) staining and glial fibrillary acidic protein (GFAP), and a sarcomatous region composed of spindle cells positive for vimentin (Vim). Frequent mitotic figures and a characteristic fascicular arrangement of cells are observed. In addition to HE staining, immunohistochemical staining for GFAP and Vim is essential for diagnosis [11].

The radiological features of GS often overlap with those of other intracranial tumors, making diagnosis based on imaging alone a significant challenge. The differential diagnosis includes: (1) GBM: Typically located in the deep white matter, GBM is characterized by significant peritumoral edema and a butterfly-shaped lesion involving the corpus callosum. It often presents with heterogeneous signals, necrosis, and cystic changes, with patchy enhancement on contrast imaging. (2) Metastatic tumors: These are frequently located at the gray-white matter junction. On MRI, metastases are hypointense on T1-weighted images and hyperintense on T2-weighted images, with thick, irregular ring enhancement after contrast administration. Extensive surrounding edema and a small lesion-to-edema ratio are common. (3) Meningioma: As an extra-axial tumor, meningioma usually has a broad Dural attachment and shows isointense or slightly hypointense T1 signals and isointense or slightly hyperintense T2 signals. Meningiomas enhance homogeneously and may exhibit the Dural tail sign, with associated bone thickening, destruction, or thinning.

Currently, there is no standard treatment protocol for GS, and its management is based on traditional GBM therapies. Maximal safe surgical resection, followed by adjuvant radiotherapy and concurrent temozolomide chemotherapy, is the mainstay of treatment [12]. The median survival time for untreated GS patients is approximately 4 months, while radiotherapy can extend the median survival to 10.6 months. Studies suggest that both radiotherapy and chemotherapy are favorable prognostic factors [13,14].

## Conclusion

This case report describes a rare instance of GS, which, compared to GBM, has a shorter clinical course and poorer prognosis. The diagnosis and treatment of GS present significant challenges due to its atypical clinical presentation, overlapping radiological features with other tumors, rarity, and propensity for extracranial metastasis. Imaging plays a critical role in determining the tumor's location, size, relationship with surrounding tissues, and detecting distant metastases. A definitive diagnosis requires a combination of imaging and histopathological analysis. Surgical resection remains the primary treatment, and adjuvant radiotherapy and chemotherapy can improve outcomes. This case underscores the importance of considering GS as a differential diagnosis when encountering intracranial neoplastic lesions, aiming to raise awareness, facilitate early diagnosis, and optimize treatment strategies to improve patient outcomes.

## Patient consent

The study involving human subject was reviewed and approved by the Medical Research Ethics Committee of Hainan General Hospital in accordance with the Helsinki Declaration. In this retrospective study, this patient's written informed consent was obtained.
